# Characterization of HIF-1α Knockout Primary Human Natural Killer Cells Including Populations in Allogeneic Glioblastoma

**DOI:** 10.3390/ijms25115896

**Published:** 2024-05-28

**Authors:** Tsutomu Nakazawa, Takayuki Morimoto, Ryosuke Maeoka, Kengo Yamada, Ryosuke Matsuda, Mitsutoshi Nakamura, Fumihiko Nishimura, Shuichi Yamada, Young-Soo Park, Takahiro Tsujimura, Ichiro Nakagawa

**Affiliations:** 1 Department of Neurosurgery, Nara Medical University, Kashihara 634-8521, Japan; t.morimoto@naramed-u.ac.jp (T.M.); r.maeoka@naramed-u.ac.jp (R.M.); k196441@naramed-u.ac.jp (K.Y.); rmatsuda@naramed-u.ac.jp (R.M.); mnaka@grandsoul.co.jp (M.N.); fnishi@naramed-u.ac.jp (F.N.); syamada@naramed-u.ac.jp (S.Y.); park-y-s@naramed-u.ac.jp (Y.-S.P.); nakagawa@naramed-u.ac.jp (I.N.); 2 Clinic Grandsoul Nara, Uda 633-2221, Japan; takahiro@grandsoul.co.jp; 3 Grandsoul Research Institute for Immunology, Inc., Uda 633-2221, Japan

**Keywords:** natural killer cell, glioblastoma, immunotherapy, hypoxia, HIF-1α, CRISPR/Cas9

## Abstract

Enhancing immune cell functions in tumors remains a major challenge in cancer immunotherapy. Natural killer cells (NK) are major innate effector cells with broad cytotoxicity against tumors. Accordingly, NK cells are ideal candidates for cancer immunotherapy, including glioblastoma (GBM). Hypoxia is a common feature of solid tumors, and tumor cells and normal cells adapt to the tumor microenvironment by upregulating the transcription factor hypoxia-inducible factor (HIF)-1α, which can be detrimental to anti-tumor effector immune cell function, including that of NK cells. We knocked out HIF-1α in human primary NK cells using clustered regularly interspaced short palindromic repeat (CRISPR)-associated protein 9 (Cas9). Then, cellular characterizations were conducted in normoxic and hypoxic conditions. Electroporating two HIF-1α-targeting guide RNA–Cas9 protein complexes inhibited HIF-1α expression in expanded NK cells. HIF-1α knockout human NK cells, including populations in hypoxic conditions, enhanced the growth inhibition of allogeneic GBM cells and induced apoptosis in GBM-cell-derived spheroids. RNA-sequencing revealed that the cytotoxicity of HIF-1α knockout NK cells could be related to increased perforin and TNF expression. The results demonstrated that HIF-1α knockout human NK cells, including populations, enhanced cytotoxicity in an environment mimicking the hypoxic conditions of GBM. CRISPR–Cas9-mediated HIF-1α knockout NK cells, including populations, could be a promising immunotherapeutic alternative in patients with GBM.

## 1. Introduction

Natural killer (NK) cells are innate immune system effectors crucial in killing abnormal cells such as tumors and virus-infected cells independently of prior sensitization. The NK-cell-killing functions are regulated by activating and inhibitory receptors on the cell’s surface, leading to the release of small granules containing perforin (PFR1) and granzymes [1,2,3,4]. Activated NK cells are cytotoxic to tumor cells and produce immunomodulatory cytokines, such as interferon (IFN)-γ and a tumor necrosis factor (TNF)-α [5]. IFN-γ is one of the most potent effector cytokines secreted by NK cells and is crucial in antiviral, antibacterial, and anti-tumor activity. IFN-γ modulates caspase-, FasL-, and TNF-related apoptosis-inducing ligand (TRAIL) expressions and activates anti-tumor immunity. Tumor stromal cells modulate effector cytokine secretion in NK cells. Signaling through activating receptor NKG2D (a C-type lectin-like receptor expressed on NK cells) promotes IFN-γ release. Interleukin (IL)-12 is produced by dendritic cells, macrophages, and neutrophils and induces IFN-γ production in NK cells, which TNF-α, IL-1, and IL-18 enhance [6,7]. Although NK cells exhibit potent anti-tumor immunity, their functionality is significantly hindered within the immunosuppressive tumor microenvironment (TME), primarily influenced by cellular and metabolic factors released by tumor cells [8,9,10]. Tumor cells use various tactics to escape NK-cell-induced elimination, directly suppressing NK-activating receptors or releasing immune-suppressive factors such as a transforming growth factor-β (TGF), IL-10, indoleamine 2,3-dioxygenase 1 (IDO1), and soluble NK receptor ligands, including UL16-binding proteins (ULBP) 1–3 and major histocompatibility complex (MHC) class I chain-related proteins (MIC) A and B [11]. Therefore, devising a therapeutic approach to restore compromised NK cell function to enhance cancer immunotherapy effectiveness is essential.

Hypoxia is a significant TME feature that consistently pervades the tumor environment due to the rapid proliferation of tumor cells without sufficient blood support caused by the inaccessibility of the oxygen source to the resident vasculature. Growing evidence suggests that hypoxia significantly influences cancer dormancy and metabolism, enhancing stemness activity and contributing to tumor initiation and cancer progression [12,13]. The transition from normoxic to hypoxic conditions during ex vivo cultivation initiates a cascade of gene transcriptional events in human cells, including NK cells [14,15]. Hypoxic conditions in the cancer microenvironment influence the NK cell phenotype, leading to tumor resistance and immune-suppressive cell production [11,16,17].

The hypoxia-inducible factor (HIF) is a transcription factor instrumental in the ability of cells to sense and adapt to oxygen level changes. HIF stabilizes immediately after exposure to hypoxia and forms a heterodimer with the nuclear protein HIF-1β/Arnt. The heterodimeric HIF-1α/β binds to the hypoxia-responsive elements to initiate the transcription of downstream target genes [18]. Several studies have demonstrated that HIF-1α expression increases in many cancers and is important in cancer progression. HIF-1α induces the expression of many genes related to cancer cell growth and survival, angiogenesis, metastasis, cancer metabolism, cancer stem cell maintenance, and resistance to cancer treatment modalities [19,20]. The role of HIF-1α in macrophages, dendritic cells, neutrophils, T cells, and B cells is as essential for cellular responses as it is in its original role in hypoxia. HIF-1α has been implicated in immunosuppression in tumors [21].

The role of HIF-1α in NK cells has been reported and summarized [22]. HIF-1α overexpression inhibited human NK cells by downregulating the NK-cell-activating receptors involved in the tumor killing [16]. HIF-1α promoted multiple signaling activities and induced immune suppression, including that of NK cells. Inhibiting HIF-1α promoted NK cell activity and inhibited tumor progression in humans and mice [23], underscoring the crucial role of HIF-1α in maintaining tumor immunosurveillance in human NK cells beyond its function as a hypoxia-responsive transcription factor. Contrastingly, specifically deleting HIF-1α in NK cells, impaired NK-cell-mediated tumor cell-killing in vitro but inhibited tumor growth in vivo in mice [24]. Thus, the results from in vitro and animal xenograft models using HIF inhibitors are therefore in sharp contrast with knockout studies, indicating a complex relationship between HIF-1α and NK phenotypes. Based on the evidence that HIF-1α inhibition promotes NK cell function, permanently inhibiting HIF-1α expression in NK cells could be a useful cancer immunotherapy modality compared to transiently inhibiting HIF-1α using drugs such as the small-molecular compound KC7F2 [23]. Clustered regularly interspaced short palindromic repeats (CRISPR) and CRISPR-associated protein 9 (Cas9) disturb the target gene via small RNAs that chaperone the Cas9 DNA nuclease to the target site through base pairing. This approach is extremely specific and efficient for engineering and disrupting eukaryotic genomes [25,26]. CRISPR/Cas9 targeting of HIF-1α could be a means of overcoming NK-cell-function-suppressive hypoxia circumstances in NK-cell-based immunotherapy for cancer, including glioblastoma (GBM).

It is now possible to expand highly purified NK cells from human peripheral blood (PB) with high efficiency [27]. This expansion technique is coupled to cell populations that have undergone CRISPR/Cas9 gene-specific knockout protocols, allowing NK phenotypes to be quantified, as demonstrated with the negative regulator gene *CIS* [28] and inhibitory receptor gene *TIM3* [29]. In the present study, we aimed to induce and expand human NK cells with HIF-1α knockout by CRISPR/Cas9. The effect of the cytotoxicity of the expanded and activated HIF-1α knockout NK cell, including populations (HIF KO NKP), against GBM cells and GBM-cell-derived spheroids under normoxic and hypoxic conditions was evaluated. Furthermore, the HIF KO NKP underwent comprehensive gene expression profiling. The results demonstrated that knocking out HIF-1α in the NK cells enhanced their inhibition of GBM cell growth in hypoxic conditions and induced apoptosis in GBM-cell-derived spheroids.

## 2. Results

### 2.1. Induction of HIF1α Knockout Human PB-Derived NK Cells

**Figure 1:**
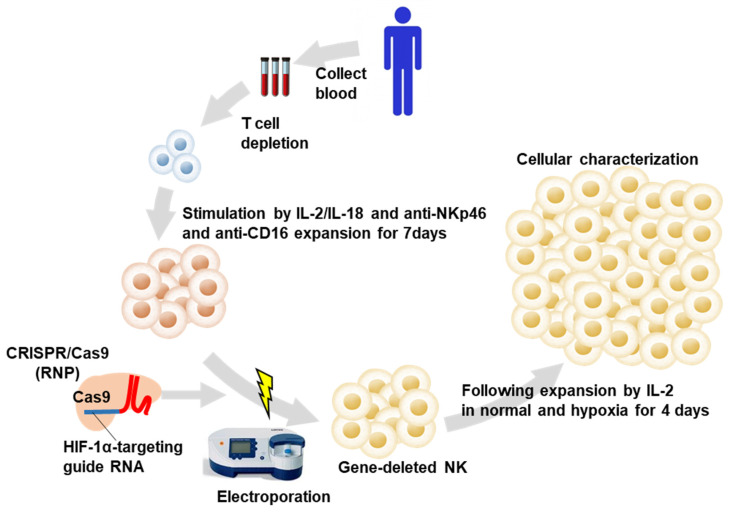
Induction and expansion of hypoxia-inducible factor (HIF)-1α knockout human primary natural killer (NK) cells.

PB mononuclear cells (PBMCs) were obtained from healthy volunteers. Figure 1 depicts the genome editing scheme of the human PB-expanded NK cells. The CD3-depleted PBMCs were cultured using IL-2, IL-18, anti-NKp46, and anti-CD16 stimulation for 7 days. The expanded cells routinely contained >90% CD3+CD56- NK cells. Ribonucleoprotein complexes (RNPs) were produced by incubating the designed singlet guide RNA (gRNA) and trans-activating crRNA (tracrRNA) with recombinant Cas9 prior to electroporation and introducing them into the in vitro expanded primary human NK cells. The NK cells were cultured for 4 days for sufficient protein turnover time (Figure 1).

**Figure 2:**
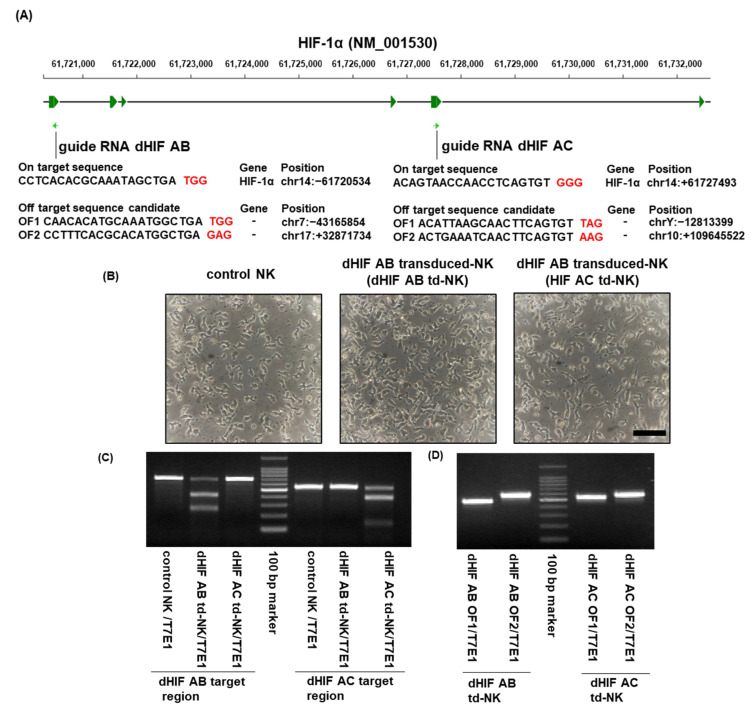

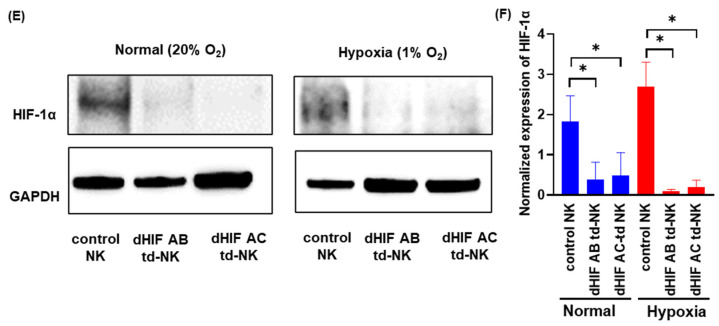
Induction and expansion of HIF1α knockout human peripheral blood (PB)-derived NK cells including populations. (**A**) Schematic representation of the HIF-1α gene (NM_001530). Green arrows indicate single guide RNA (sgRNA) targeting sites. Two sgRNAs (dHIF AB and dHIF AC) were designed. Top and bottom tables: On-target (OT) sequences and predicted off-target (OF) sequences of the HIF-1α-targeting gRNAs, respectively. Red letters in the target sequences: Protospacer adjacent motif (PAM) sequence. Gene name and genome positions are presented on the right side of the sequences. (**B**) Inverted microscope morphology of sgRNA-transduced NK cells electroporated after 4 days. Left: Contro NK cells, middle: dHIF AB-transduced NK cells (dHIF AB td-NK cells), right: dHIF AC td NK cells. Black bar = 100 μm. (**C**) OT effects in the genome-edited NK cells. NK cell genomic DNA was isolated, and PCR was performed using primers flanking the OT regions. The PCR product was reacted with T7 endonuclease 1 (T7E1). The three lanes on the left and right side of the 100 bp marker (center) indicate the T7E1-digested PCR product amplified using the dHIF AB and dHIF AC flanking region primers, respectively. (**D**) OF detection by T7E1 assay. PCR was performed using primers flanking the OF region. The OF sites were predicted using an OF potential checking system based on a homology-based algorithm (see Section 4). The top two expected sites were analyzed. The two lanes on the left and right of the 100 bp marker (center) contain the PCR products of the dHIF AB and dHIF AC OF region, respectively. (**E**) Representative HIF-1α protein expression in genome-edited NK cells expanded from human PB. Top and bottom blots: HIF-1α protein and GAPDH protein (internal control), respectively. gRNA-electroporated NK cells were additionally cultured for 4 days (total 11-day culture) in normoxic (20% O_2_, left) and hypoxic conditions (1% O_2_, right). (**F**) Normalized expression of HIF-1α. The relative HIF-1α expression was measured by densitometry. The HIF-1α intensity was divided by the GAPDH expression intensity. Data are the mean ± SD. Statistical differences were determined by one-way analysis of variance (ANOVA) following Sidak’s multiple comparison test. At least two independent experiments were performed. n = 3, * *p* < 0.01.

Two guide RNAs (gRNAs) targeting the HIF-1α gene were designed (Figure 2A). The experiments were performed according to the scheme in Figure 1. ‘Control NK cells’ were NK cells that had undergone a mock transformation, and we then used the term ‘control NK cells’ throughout. The RNP electroporation and transduction demonstrated no morphological changes on day four after electroporation in individual cellular populations (Figure 2B). The predicted on-target (OT) and off-target (OF) effects were detected with a T7 endonuclease 1 (T7E1)-based mutation detection assay on the RNP-electroporated NK cells on day four after electroporation. Figure 2A presents the OT and OF site sequences. The assays demonstrated that both gRNAs cleaved the HIF-1α target gene region (Figure 2C), demonstrating the effective OT effects. The OF sites were predicted with a homology-based OF-targeting potential detection system using an online tool (Section 4, and Figure 2A). The assays demonstrated that both gRNAs did not exert the predicted OF effects (Figure 2D). Western blotting revealed that hypoxia tended to upregulate HIF-1α expression in controlled NK cells more than in the normoxic conditions. The transduction of both sgRNAs inhibited HIF-1α expression almost completely in normoxic and hypoxic conditions (Figure 2E,F). The results demonstrated the efficient induction of HIF KO NKP.

### 2.2. Anti-Tumor Effects of HIF-1α Knockout Human Primary NK Cells Including Populations on Allogeneic GBM Cells in Normoxic and Hypoxic Conditions

**Figure 3:**
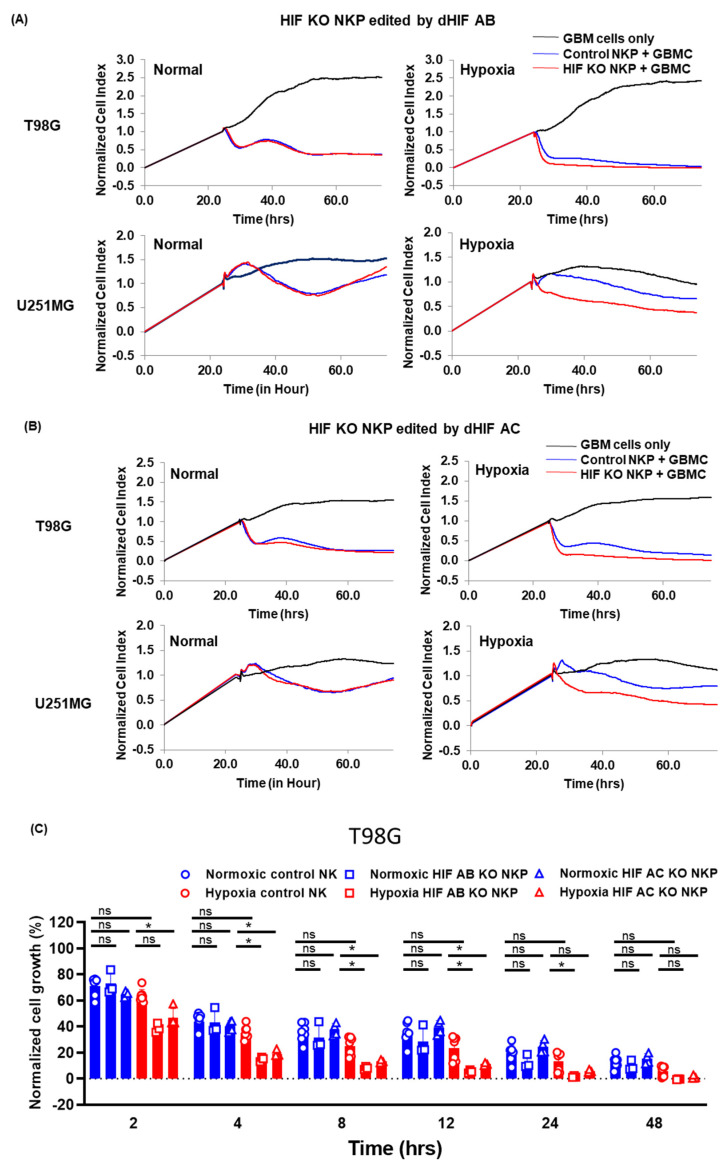

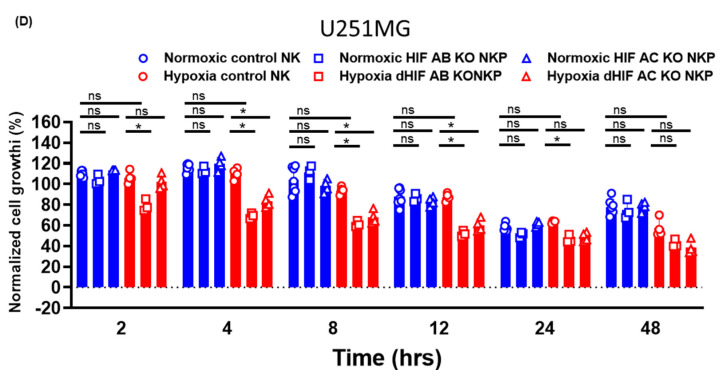
Growth inhibition effects of HIF-1α knockout human primary NK cells including populations on allogeneic glioblastoma cells (GBMC) in normoxic and hypoxic conditions. (**A**) Representative graph of real-time growth curve of T98G (top) and U251MG cells (bottom) co-cultured with control NK cell population (NKP; blue) or HIF AB knockout NK cells including population (HIF AB KO NKP; red) at effector-to-target (E:T) cell ratios of 0.5:1. The growth curve (black) indicates GBM cell lines only. Left and right graphs depict normoxic and hypoxic conditions, respectively. (**B**) Representative graph of real-time growth curve inhibition of T98G (top) and U251MG cells (bottom) co-cultured with control NK cells (blue) or HIF AC KO NKP (red) at E:T cell ratios of 0.5:1. Left and right graphs show normoxic and hypoxic conditions, respectively. (**C**,**D**) Graphs depicting the real-time growth inhibition assays of T98G (**C**) and U251MG cells (**D**). Blue circles: control NK cells, blue squares: HIF AB KO NKP, blue triangles: HIF AC KO NKP in normoxic conditions. Red circle: control NKP, red squares: HIF AB KO NKP, red triangles: HIF AC KO NKP in hypoxic conditions. *x*- and *y*-axes: Normalized cell growth and incubation duration, respectively. Data are the mean ± SD of 3–7 experiments. At least two independent experiments were performed. Statistical differences were determined by two-way ANOVA, followed by Tukey’s test. * *p* < 0.05, ns: not significant.

The functional aspects of the HIF KO NKP were evaluated using cell lines derived from allogeneic GBM cells in vitro through real-time cell analyzer (RTCA)-based growth inhibition assays. In normoxic conditions (20% O_2_), the control NK cells, HIF AB knockout NK cells including a population (HIF AB KO NKP), and HIF AC KO NKP significantly inhibited the T98G and U251MG GBM cell growth time dependently compared to the target GBM cells only. Compared to control NK cells, HIF AB and AC KO NKP did not significantly alter the growth inhibition of the GBM cells (Figure 3A–D). In hypoxic conditions (1% O_2_), HIF AB and AC KO NKP significantly inhibited T98G and U251MG cell growth time dependently compared to the target cells. Interestingly, HIF AB and AC KO NKP significantly inhibited the growth inhibition of the GBM cells compared to control NK cells in hypoxia (Figure 3A–D). Furthermore, the growth inhibition of the control NK cells did not significantly alter the GBM cell growth inhibition in normoxic and hypoxic conditions. The results indicated that HIF KO NKP enhanced the cytotoxicity-mediated GBM cell growth inhibition in hypoxic conditions but not normoxic conditions.

### 2.3. HIF-1α Knockout Human NK Cells Including Populations Enhances Apoptosis Induction of Spheroids Derived from Allogeneic GBM Cells in Hypoxic Conditions

**Figure 4:**
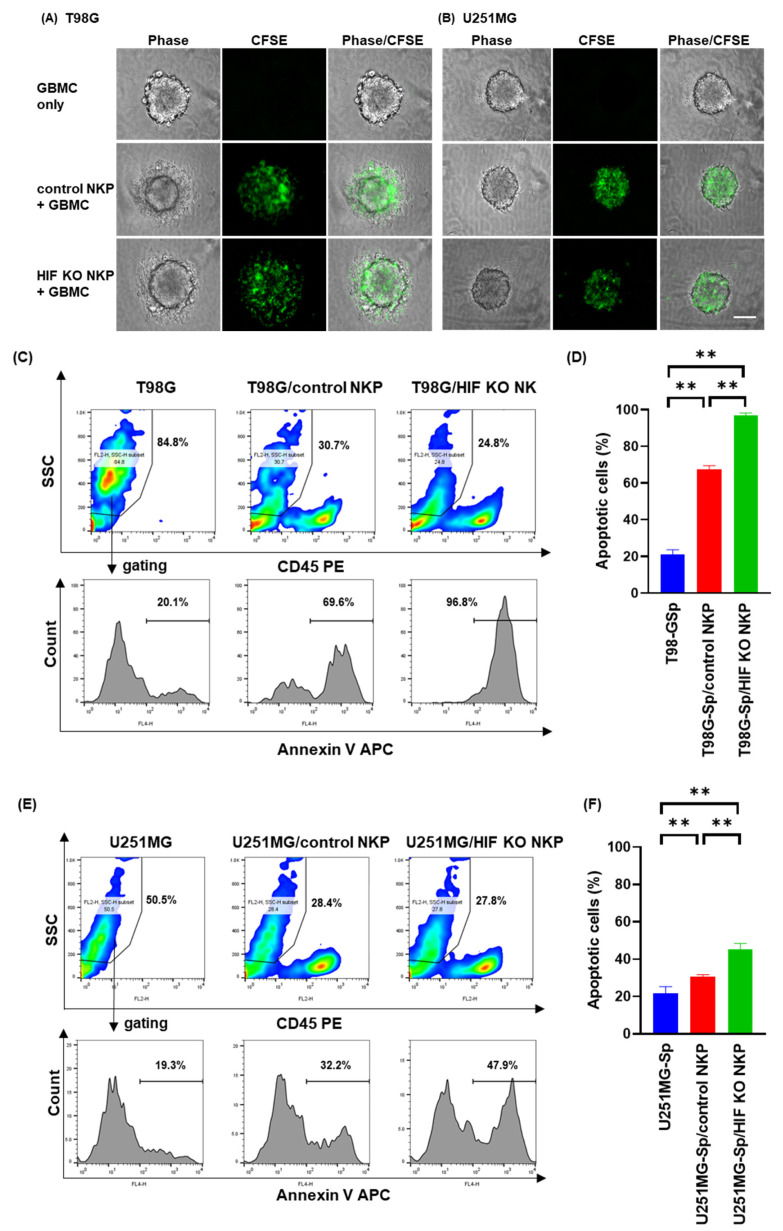
Apoptosis induction effects of HIF-1α knockout human NK cells including populations on spheroids derived from allogeneic GBM cells in hypoxic conditions. (**A**,**B**) Fluorescent microscopic evaluation of (**A**) T98G- and (**B**) U251MG-derived spheroids co-cultured with fluorescent-labeled NK cells. White scale bar = 100 μm. GBM cells (300 cells/well) were seeded onto nonadherent V-bottom 96-well plates for 1 day, co-cultured with 3 × 10^3^ NK cells, and observed under a BZ-X700 fluorescence microscope. The cells in all spheroids were visualized by recording merged Z-stack images using the BZ-X700 quick full-focus function. Phase contrast (left, Phase), FL1-based fluorescent (middle, CFSE), and overlay (right, Phase/CFSE) images are shown. (**C**,**E**) Representative flow cytometric data of the apoptosis of (**C**) T98G- and (**E**) U251MG-derived spheroids co-cultured with NK cells. The spheroids (5 × 10^3^) were co-cultured for 24 h with 5 × 10^4^ NK cells. Subsequently, the cells were centrifuged and detached, then stained with allophycocyanin (APC)-conjugated annexin V and phycoerythrin (PE)-conjugated CD45. Apoptotic GBM cells were detected with a flow cytometer. Analysis accuracy was ensured by gating out the CD45-positive fraction to assess GBM cell apoptosis. Top panels: Density plots of SSC/CD45 PE. Red and blue color maps: High and low density, respectively. Bottom panels: Histograms of annexin V-positive cells negatively fractionated by FL-2-positive cells (CD45-PE). *x*-axis: APC-conjugated annexin V. (**D**,**F**) Graphs indicating the percentage of annexin V-positive (**D**) T98G- and (**F**) U251-derived GBM cells. Data are the mean ± SD (n = 4). At least two independent experiments were performed. Statistical differences were determined by one-way ANOVA, followed by Tukey’s test. ** *p* < 0.01.

We assessed the apoptosis induction effects of the NK cells on GBM spheroids through flow cytometry to confirm the enhanced cytotoxicity of HIF KO NKP in hypoxic conditions. Fluorescent microscopic analysis of the NK cell dynamics on GBM spheroids revealed NK cell accumulation around the spheroids. We did not observe distribution changes in HIF KO NKP (Figure 4A,B). Annexin V-positive apoptotic cells in the GBM spheroids were analyzed by gating out the CD45-positive fractions. The control NK cells and dHIF NK cells respectively induced 64.8–69.6% and 95.5–98.0% annexin V-positive cells among the T98G cells and 29.7–32.2% and 40.9–47.9% annexin V-positive cells among the U251MG cells. The HIF KO NKP induced significantly higher GBM spheroid apoptosis compared to the control NK cells (Figure 4D,F). The results support the idea that disrupting HIF-1α is critical for enhancing NK cell activity against GBM spheroids in hypoxic conditions.

### 2.4. RNA-Sequencing (RNAseq) Analysis of HIF-1α Knockout NK Cells Including Populations in Hypoxic Conditions

**Figure 5:**
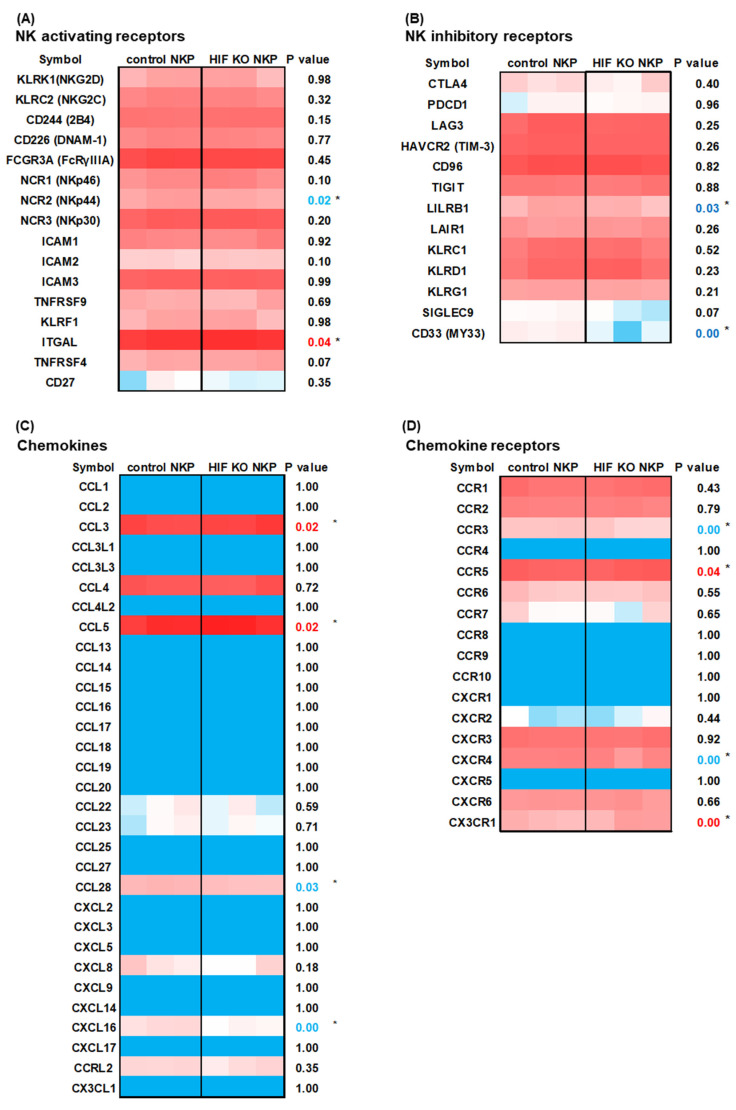

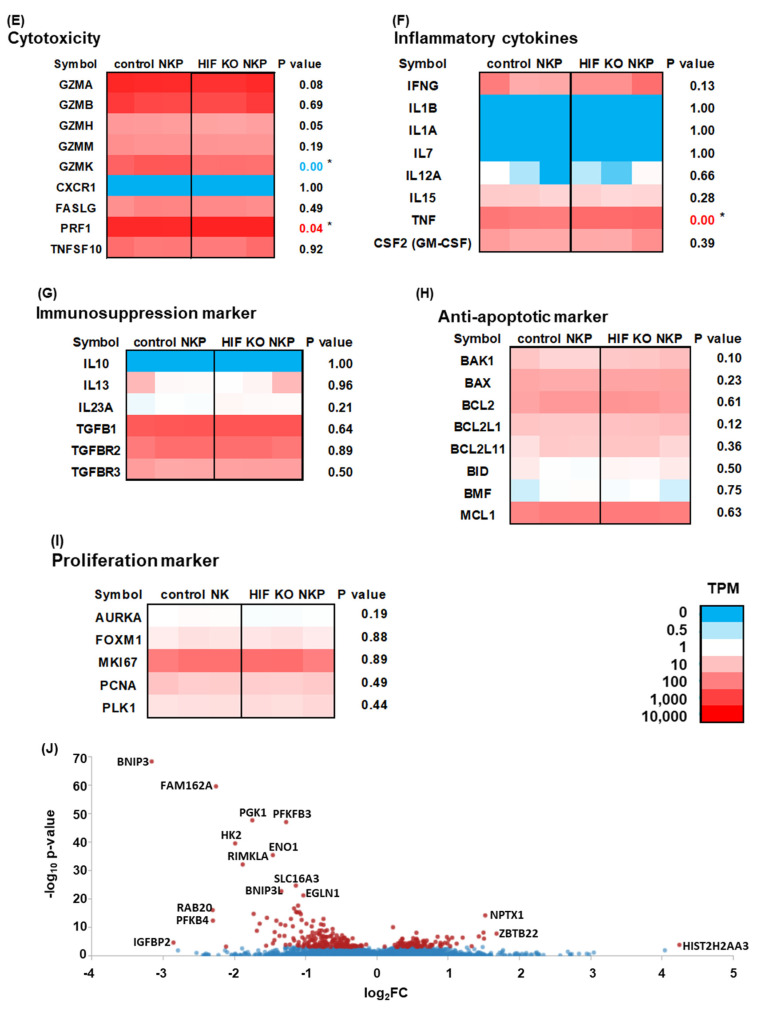

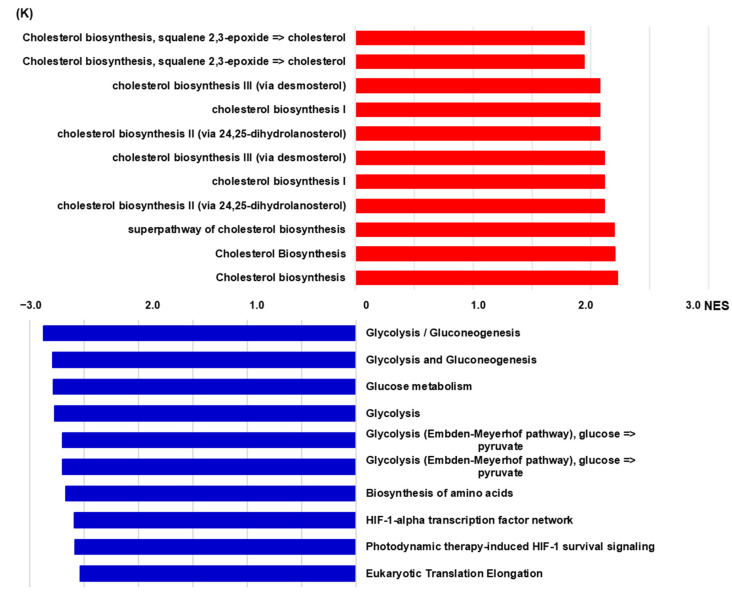
RNA-sequencing (RNAseq)-based comprehensive gene expression analysis of HIF-1α knockout human primary NK cells including populations in hypoxic conditions. (**A**–**I**) Heat maps representing genes clustered in NK-activating receptors (**A**), NK inhibitory receptors (**B**), chemokines (**C**), chemokine receptors (**D**), cytotoxicity (**E**), inflammatory cytokines (**F**), immune suppression markers (**G**), anti-apoptosis markers (**H**), and proliferation markers (**I**) with scaled intensities under GOBP (Gene Ontology Biological Process). These genes are referenced in [28]. Red and blue tones: Higher and lower gene expression, respectively, which reflects transcripts per kilobase million (TPM). The significance of differences was determined using the *t*-test. n = 3, * *p* < 0.05. Red and blue *p*-values: Upregulated and downregulated genes, respectively. All data were from at least two independent experiments. (**J**) Volcano plot analysis. *x*- and *y*-axes: log_2_ fold change (FC) and log_10_ *p*-value, respectively. Red dots: *p* < 0.05. Marked gene expression changes are labeled. (**K**) Gene set enrichment analysis (GSEA). NES = Normalized enrichment score. GSEA normalizes the enrichment score to account for differences in gene set size and correlations between gene sets and the expression dataset. Selected *p*-values are *p* < 0.01. Red and blue bars: Upregulated and downregulated gene pathway sets, respectively. The data were computed using RaNAseq (https://ranaseq.eu/index.php, accessed on 1 December 2023).

The control NK cell versus HIF KO NKP transcriptomes in hypoxia were profiled to identify differentially expressed genes (DEGs). The expressions of the following NK cell immunity-related genes were assessed: NK-activating receptors, NK inhibitory receptors, chemokines, chemokine receptors, cytotoxicity markers, inflammatory cytokines, immunosuppressive effectors, anti-apoptotic markers, and proliferation markers. These genes were selected from our previously published study [28]. Among the HIF KO NKP in hypoxic conditions, *NCR2* (NKp44) was downregulated and *ITGAL* was upregulated significantly (Figure 5A); *LILRB1* and *CD33* were significantly downregulated (Figure 5B); *CCL2* and *CCL5* were upregulated and *CCL28* and *CXCL16* were downregulated (Figure 5C); *CCR5* and *CX3CR1* were upregulated and *CCR3* and *CXCR4* were downregulated (Figure 5D); *GZMK* (granzyme K) was downregulated, while *PRF1* was upregulated (Figure 5E); *TNF* was upregulated (Figure 5F). Among the HIF KO NKP, the immunosuppression, anti-apoptosis, and proliferation genes were not significantly upregulated or downregulated (Figure 5G–I). Supplementary Table S1 lists the raw data.

Volcano plots of the DEGs demonstrated that HIF KO NKP markedly downregulated *BNIP3*, *FAM162A*, *PGK1*, *PFKFB3*, *HK2*, *ENO1*, *RIMKLA*, *SLC16A3*, *BNIP3L*, *SLC16A3*, *EGLN1*, *RAB20*, *PFKB4*, and *IGFBP2*, while *HIST2H2AA3*, *NPTX1*, and *ZBTB22* were markedly upregulated (Figure 5J). Supplementary Table S2 lists the raw data.

Gene set enrichment assays (GSEA) revealed that the cholesterol biosynthesis-related pathways were significantly upregulated in HIF KO NKP, while the glycolysis, biosynthesis of amino acids, HIF-1α networks, and eukaryotic translation elongation pathways were downregulated (Figure 5K). Supplementary Table S3 lists the raw data.

## 3. Discussion

A higher number of tumor-infiltrating NK cells is associated with better survival benefits in solid tumors [30,31,32]. However, NK cells in solid tumors exhibit an inactive and dysfunctional state [8,33]. It was reported that GBM rarely included CD3-CD56+ NK cells [34]. Our goal was to trigger an anti-tumor effect by the direct administration of NK cells involved in early immunity into immunologically cold GBM where NK cells are almost non-existent. Novel strategies to induce activated NK cells in the TME are essential for improving current NK-cell-based immunotherapy. Providing long-lived activated NK cells in the TME would achieve a stronger anti-tumor effect.

HIF-1 is an instrumental transcriptional factor in the cell’s ability to sense and adapt to oxygen level changes. The present study demonstrates that HIF-1α has an immune checkpoint function that inhibits NK cell function in hypoxic conditions that mimic the GBM microenvironment. The lack of HIF-1α in NK cells resulted in high cytotoxicity against allogeneic GBM cells and spheroids, which might be reflected in elevated TNF and the cytotoxicity-related gene PRF1 in human NK cells. A similar study by Ni et al. on a mouse model and human leukemic cells strongly supported our data. The authors used KC7F2 to temporarily inhibit HIF-1α in human NK cells [23]. In the present study, we permanently inhibited HIF-1α expression using genome editing.

We demonstrated that the activated and expanded NK cells expressed HIF-1α in normoxic cell culture conditions (20% O_2_). The hypoxic (1% O_2_) condition did not significantly alter HIF-1α expression but tended to enhance it. Cluff et al. characterized HIF-1α expression and function in expanded human primary NK cells using antibodies against the NK-cell-activating receptors NKp46 and CD2, supplemented with 400 IU/mL IL-2. The expanded NK cells did not express HIF-1α, but HIF-1α protein expression was robust under hypoxic conditions. Importantly, following expansion, the HIF-1α protein was no longer detected when IL-2 was depleted in the cells, confirming the requirement of IL-2 for HIF-1α expression. Furthermore, the dependence of the expanded NK cells on the PI3K–mTOR pathway for HIF-1α expression was confirmed [35]. Our study differed from that of Cluff et al. on HIF-1α expression in expanded NK cells under normoxic culture conditions. We believe that the difference might depend on the IL-2 concentration in the NK cell expansion. We expanded the NK cells using anti-NKp46 antibodies and extremely-high-concentration IL-2 (3000 IU/mL). The high-concentration IL-2 could stimulate PI3K–mTOR and restore HIF-1α expression in the NK cells under normoxic culture conditions.

Our data demonstrate that the loss of HIF-1α enhanced the NK-cell-killing-mediated GBM cell growth inhibition in the hypoxia environment but did not affect growth inhibition under normoxic culture conditions. The data suggest that HIF-1α strongly suppresses the mechanism by which NK-cell-killing is enhanced under hypoxia. Our RNAseq analysis of the genes regulated in NK cells in hypoxia compared to in normoxic conditions demonstrates that hypoxic exposure upregulated *FOSB*, *FFAR3*, *CYP26A1*, *CREB3L3*, *FOS*, and *C15orf48* (log_2_ fold change [FC] > 3, *p* < 0.05), while downregulating *ABCG1*, *ASS1*, and *CBS* (log_2_FC < −3, *p* < 0.05) (Supplementary Table S4). The data indicate that the genes strongly associated with cytotoxicity could not be identified. GSEA demonstrates that the DNA and RNA metabolism-related terms were increased in hypoxia compared to normoxic conditions (Supplementary Table S5 and Figure S1). Nuclear acid synthesis was, at least, activated in the NK cells in hypoxic conditions, and inhibiting HIF-1α expression in this environment enhanced killing by the NK cells.

HIF-1α/β initiated the transcription of downstream target genes, including B-cell lymphoma 2 (BCL2)-interacting protein 3 (*BNIP3*), lactate dehydrogenase A (*LDHA*), pyruvate dehydrogenase kinase isoform 1 (*PDK1*), and vascular endothelial growth factor (*VEGF*) [18]. Hypoxic conditions upregulated glycolytic enzymes such as LDHA and mitochondrial function inhibitors such as PDK1 and BNIP3, facilitating a metabolic shift from oxidative phosphorylation to glycolysis. This metabolic adaptation is a hallmark shared with stem cells [36] and long-lived NK cells [37]. Additionally, BNIP3 induced mitophagy, reducing mitochondrial function [38]. PDK1 deactivated pyruvate dehydrogenase (PDH), impeding pyruvate conversion to acetyl-CoA and suppressing the tricarboxylic acid (TCA) cycle in mitochondria [39]. Our data demonstrate that *BNIP3*, *LDHA*, *PDK1*, and *VEGF* were downregulated (log_2_FC < −1, *p* < 0.05, Figure 5J and Supplementary Table S4) and validated the RNAseq data. The DEG data demonstrate that the necrosis-inducible cytokine TNF and the cytotoxicity-related granule PFR1, a pore-forming cytolytic protein that collaborates with the cytotoxic granule granzyme for cytotoxicity, were upregulated in the HIF KO NKP (Figure 5E). TNF and PRF1 could be related to the increased cytotoxicity of NK cells in hypoxic conditions. Further analysis would be needed to demonstrate the mechanism by which TNF and PFR1 expression increases.

The HIF-1α knockout NK cells, including populations, were preclinically evaluated using an ex vivo 3D spheroid model derived from GBM cell lines. In the model, HIF KO NKP induced GBM cell death compared to unmodified NK cells. Three-dimensional cell culture techniques, including spheroid models, have expanded the possibilities for creating more physiologically relevant human cancer models. These advanced preclinical models are crucial for improving fundamental cancer research translation into new treatment approaches that benefit patients with cancer [40]. We previously reported that the spheroid model demonstrated enriched cell growth, a progression pathway, and anti-cancer therapeutic resistance. The model resembles the complexity of different healthy and diseased human tissues more closely compared to the 2D cell culture model [41]. Our data indicate that the HIF KO NKP could be an effective and feasible cell-based immunotherapy for GBM.

## 4. Materials and Methods

### 4.1. Ethics

This study was conducted according to Nara Medical University guidelines. All procedures that involved human participants were conducted according to institutional and/or national research committee ethical standards and the 1964 Declaration of Helsinki and its subsequent alterations or equivalent ethical standards.

### 4.2. Cell Lines

T98G and U251MG GBM cells were from RIKEN BioResource Center (Tsukuba, Japan) and JCRB Cell Bank (Osaka, Japan), respectively. The cell lines were authenticated and tested as mycoplasma-free. The cells were maintained in Dulbecco’s modified Eagle’s medium (DMEM; Life Technologies, Carlsbad, CA, USA) containing 100 μg/mL streptomycin, 100 U/mL penicillin (Thermo Fisher Scientific, Waltham, MA, USA), and 10% heat-inactivated fetal bovine serum (FBS; MP Biomedicals, Tokyo, Japan) at 37 °C in a humidified atmosphere with 5% CO_2_.

### 4.3. Single Guide RNAs

Two sgRNAs targeting the human HIF-1α gene locus were designed according to the manufacturer’s instructions (IDT, Coralville, IA, USA, https://sg.idtdna.com/site/order/designtool/index/CRISPR_PREDE SIGN, accessed on 1 May 2022). The targets were selected based on high OT potential values (>60%) and low OF risk values (>80%) calculated by the IDT algorithm. Subsequently, the targets without exon regions in the top five OF algorithm predictions were selected. The HIF-1α target sequences dHIF AB and dHIF AC were CCTCACACGCAAATAGCTGATGG (chromosome 14: 61720556–61720534) and ACAGTAACCAACCTCAGTGTGGG (chromosome 14: 61727493–61727515), respectively. The underlined sections indicate the protospacer adjacent motif (PAM) sequence. The negative control sgRNA was from IDT, and the sequence is as follows: rCrGrUrUrArArUr-CrGrCrGrUrArUrArArUrArCrGrGrUrUrUrUrArGrAr-GrCrUrArUrGrCrU.

### 4.4. Induction of HIF-1α Knockout NK Cells Including Populations from Human PB

The highly purified human NK cells were expanded as previously described [27]. Briefly, PBMCs were obtained from 16 mL heparinized peripheral blood from two healthy male volunteers (47 and 43 years old). The PBMC CD3 fraction was depleted using RosetteSep™ Human CD3 Depletion Cocktail (STEMCELL Technologies, Vancouver, BC, Canada). The CD3-depleted PBMCs (2 × 10^6^ cells) were placed for 7 days in a 6-well plate (Corning, Steuben, NY, USA) coated with anti-NKp46 (clone 195314, R&D Systems, Minneapolis, MN, USA) and anti-CD16 antibody (clone 3G8, Thermo Fisher Scientific) (both 5 µg/mL) and containing 2 mL AIM-V medium (Life Technologies) supplemented with 50 ng/mL recombinant human IL-18 (rhIL-18, Medical & Biological Laboratories Co., Ltd., Nagoya, Japan), 10% autologous plasma, and 3000 IU/mL rhIL-2 (Novartis, Basel, Switzerland) at 37 °C in a humidified atmosphere with 5% CO_2_. The AIM-V medium containing 3000 IU/mL rhIL-2 was refilled as required. Genome ed iting was conducted as previously described with minor modifications [28,29]. Expanded NK cells (3 × 10^6^) were electroporated to RNP complexes (targeted sgRNA/tracrRNA and recombinant Cas9, IDT) using a Human NK Cell Nucleofector Kit (VPA-1005; Lonza, Basel, Switzerland) and electroporation program X-001. Subsequently, the cells were resuspended in AIM-V medium containing 10% autologous plasma and 3000 IU/mL rhIL-2 and placed for 4 days in a 12-well plate (Corning) at 37 °C in a humidified atmosphere with 5% CO_2_ and 20% or 1% O_2_.

### 4.5. Efficacy of CRISPR/Cas9 Gene Disruption

The genome-edited NK cells were harvested 4 days after electroporation, their DNA was extracted using a QIAamp DNA mini kit (Qiagen, Hilden, Germany), and T7E1 mismatch detection assays were conducted using an Alt-R Genome Editing Detection Kit (IDT) as described previously [28,29,42]. Briefly, the OT and OF sites and adjacent sequences from the genomic DNA were amplified using KOD FX enzyme solution (TOYOBO, Osaka, Japan). The PCR conditions were as follows: one cycle at 94 °C for 2 min, followed by 40 cycles at 98 °C for 10 s, 63 °C for 30 s, and 68 °C for 30 s, and a final cycle at 68 °C for 7 min. The PCR was performed using a LifeECO thermal cycler (Bioer Technologies Co., Ltd., Hangzhou, China). The PCR primers were from Thermo Fisher Scientific. The subsequent PCR was performed on the LifeECO thermal cycler with the following cycling conditions: 95 °C for 5 min, decrease from 95 °C to 85 °C at a rate of 2 °C per second, decrease from 85 °C to 25 °C at a rate of 0.1 °C per second, followed by a decrease to 4 °C until electrophoresis. The rehybridized PCR products were digested for 30 min using T7E1 and separated for 20 min on 2% agarose gel. The DNA was visualized under a UV transilluminator (FAS-IV, Nippon Genetics Co., Ltd., Tokyo, Japan). OF mutagenesis, predicted using a gene homology-based OF potential checking system (IDT), was detected in the same manner. Supplementary Table S6 lists the PCR primers used to amplify the target locus.

### 4.6. RNAseq

RNAseq analysis was performed as described previously [43]. Briefly, the total RNA of genome-edited NK cells 4 days after electroporation was extracted using NucleoSpin RNA (Takara Bio, Shiga, Japan). The total RNA underwent next-generation sequencing at Amelieff (Tokyo, Japan). The sequencing libraries were generated using the NEBNext^®^ Ultra™ RNA Library Prep Kit for Illumina^®^ (NEB, Ipswich, MA, USA, Vat No. 7760) following the manufacturer’s protocol and purified using the AMPure XP system (Beckman Coulter, Brea, CA, USA). Library quality was assessed using the Agilent Bioanalyzer 2100 system (Agilent, Santa Clara, CA, USA). Sequencing was performed using the NovaSeq 6000 (Illumina, San Diego, CA, USA) with a 150 bp end read setting. Reads were aligned to the *Homo sapiens* reference genome (hg38) using RNA STAR. The read counts per gene were obtained using featureCounts (version 2.0.0). The relative expression level of each gene was normalized by the transcripts per million (TPM). The genetic characteristics were analyzed and visualized using RaNAseq (https://ranaseq.eu/index.php, accessed on 1 December 2023) [44].

### 4.7. Western Blotting

Western blotting was conducted as previously described [28]. Briefly, 10^6^ NK cells were dissolved in radioimmunoprecipitation assay lysis and extraction buffer with Halt protease inhibitor cocktail (Thermo Fisher Scientific) and sonicated using the ultrasound-based Sonifier 250 homogenizer (Branson, Hannover, Germany) according to the manufacturer’s instructions. The whole-cell lysate was mixed with 4× Bolt LDS sample buffer (Thermo Fisher Scientific) and incubated for 10 min at 70 °C. Subsequently, 4–12% sodium dodecyl sulfate–polyacrylamide gel electrophoresis was conducted using 5 μL (for glyceraldehyde-3-phosphate dehydrogenase [GAPDH]) to 40 μL (for HIF-1α) lysate, then the blots were transferred onto a PVDF membrane using the iBlot 2 Dry Blotting System (Thermo Fisher Scientific). The membranes were reacted at room temperature using an iBind Automated Western System (Thermo Fisher Scientific).

The primary antibodies were rabbit polyclonal IgGs against HIF-1α (1:500 dilution, clone D1S7W, Cell Signaling, Danvers, MA, USA) and GAPDH (1:1000 dilution, clone 14C10, Cell Signaling). The secondary antibody was horseradish peroxidase-conjugated anti-rabbit IgG (1:200 dilution, Cell Signaling). The blots were developed using SuperSignal West Pico PLUS Chemiluminescent Substrate (Thermo Fisher Scientific). The signal intensity was determined using FUSION Solo 7S Edge (Vilber Bio Imaging, Paris, France).

### 4.8. Growth Inhibition Assays

The inhibitory effects of the genome-edited NK cells on GBM cells were examined using xCELLigence RTCA (real-time cell analysis) S16 and DP instruments (ACEA Biosciences, San Diego, CA, USA) in electrical impedance-based assessment as described previously [28,29,42]. Briefly, 100 μL complete medium was added to each well on an E-plate 16 (ACEA Biosciences). The background impedance was measured at 37 °C in a humidified atmosphere with 5% CO_2_. The GBM cells (2 × 10^4^ T98G or U251MG cells/well) were seeded as the target (T) cells, and the impedance was recorded every 15 min for 24 h. After 24 h, the genome-edited NK cells were added to each well as effector (E) cells in E:T cell ratios of 1:1, and the impedance was recorded every 5 min for 48 h. The data were analyzed using RTCA version 1.2 (ACEA Biosciences), and the normalized cell growth was calculated.

### 4.9. GBM Spheroid Invasion Assay

Fluorescence microscopy analysis was conducted as described previously [28]. Briefly, 5 × 10^5^ NK cells were suspended with 1 µg/mL carboxyfluorescein diacetate succinimidyl ester (CFSE; Dojindo Laboratories, Kumamoto, Japan) and incubated for 30 min at 37 °C. The spheroids derived from 300 T98G and U251MG cells were co-cultured with 1250 CFSE-labeled NK cells for 24 h and observed under a BZ-X700 all-in-one fluorescence microscope (Keyence, Osaka, Japan). The CFSE-labeled NK cells were detected using the green fluorescent protein filter (OP-87765, Keyence). The cells within all spheroids were visualized by recording merged Z-stack images using the BZ-X700 quick full-focus function.

### 4.10. Cytotoxicity Assays for Spheroids

The flow cytometry-based apoptosis assay for spheroids was conducted as previously described [28] and slightly modified. Briefly, the spheroids derived from 3 × 10^3^ GBM cells were co-cultured with 1.5 × 10^4^ NK cells for 24 h. Then, the cells were centrifuged and detached for 60 min at 37 °C using StemPro Accutane Cell Dissociation Reagent (Thermo Fisher Scientific). Subsequently, the cells were stained with phycoerythrin (PE)-labeled anti-CD45 antibody (HI30, BioLegend, San Diego, CA, USA) and an allophycocyanin (APC)-conjugated Annexin V Apoptosis Detection Kit according to the manufacturer’s instructions (BioLegend). Apoptotic GBM cells were detected using a BD FACSCalibur flow cytometer (BD Biosciences, San Jose, CA, USA) and analyzed using FlowJo v10 (BD Biosciences).

### 4.11. Statistical Analysis

The statistical analysis was performed using GraphPad Prism 8 (GraphPad Software, San Diego, CA, USA). The values are reported as the mean ± SD. The statistical significance of differences was determined using the unpaired *t*-test, Mann–Whitney test, or one- or two-way analysis of variance (ANOVA), followed by Tukey’s or Sidak’s test. Statistical significance was accepted at *p* < 0.05.

## 5. Conclusions

HIF-1α knockout by CRISPR/Cas9 in NK cells enhanced the cytotoxicity in an environment mimicking the hypoxic conditions of allogeneic GBM. Allogeneic HIF-1α knockout NK cells, including populations, might be a promising immunotherapeutic alternative for patients with GBM.

## Data Availability

The datasets generated during the current study are available from the corresponding author upon reasonable request. All transcriptomics data is deposited Sequence Read Archive (SRA), accession number PRJNA1077720. A link to SRA dataset will also be available at https://www.ncbi.nlm.nih.gov/sra, accessed on 25 February 2024.

## Supplementary Materials

The following supporting information can be downloaded at https://www.mdpi.com/article/10.3390/ijms25115896/s1.
