# Supplementary material for: Characterization of HIF-1α Knockout Primary Human Natural Killer Cells Including Populations in Allogeneic Glioblastoma
Source: Int J Mol Sci. 2024 May 28;25(11):5896. doi: 10.3390/ijms25115896 (PMC11173110; doi:10.3390/ijms25115896)
Supplement: Supplementary file 1 [file ijms-25-05896-s001.zip › Supplementary Figure S1.pdf]

(A)

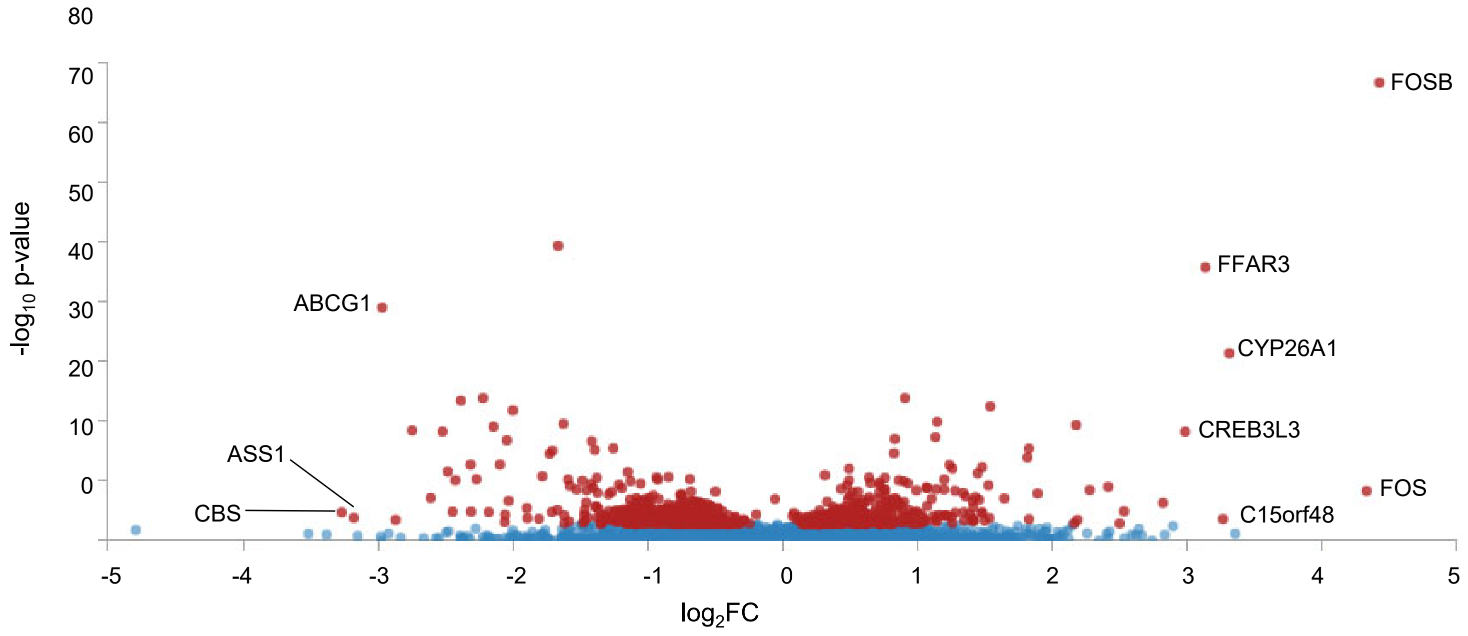

80
70
60
50
40
30
20
10
0
-log10 p-value
FOSB
FFAR3
ABCG1
CYP26A1
CREB3L3
ASS1
FOS
CBS
C15orf48
-5
-4
-3
-2
-1
0
1
2
3
4
5
log2FC

(B)

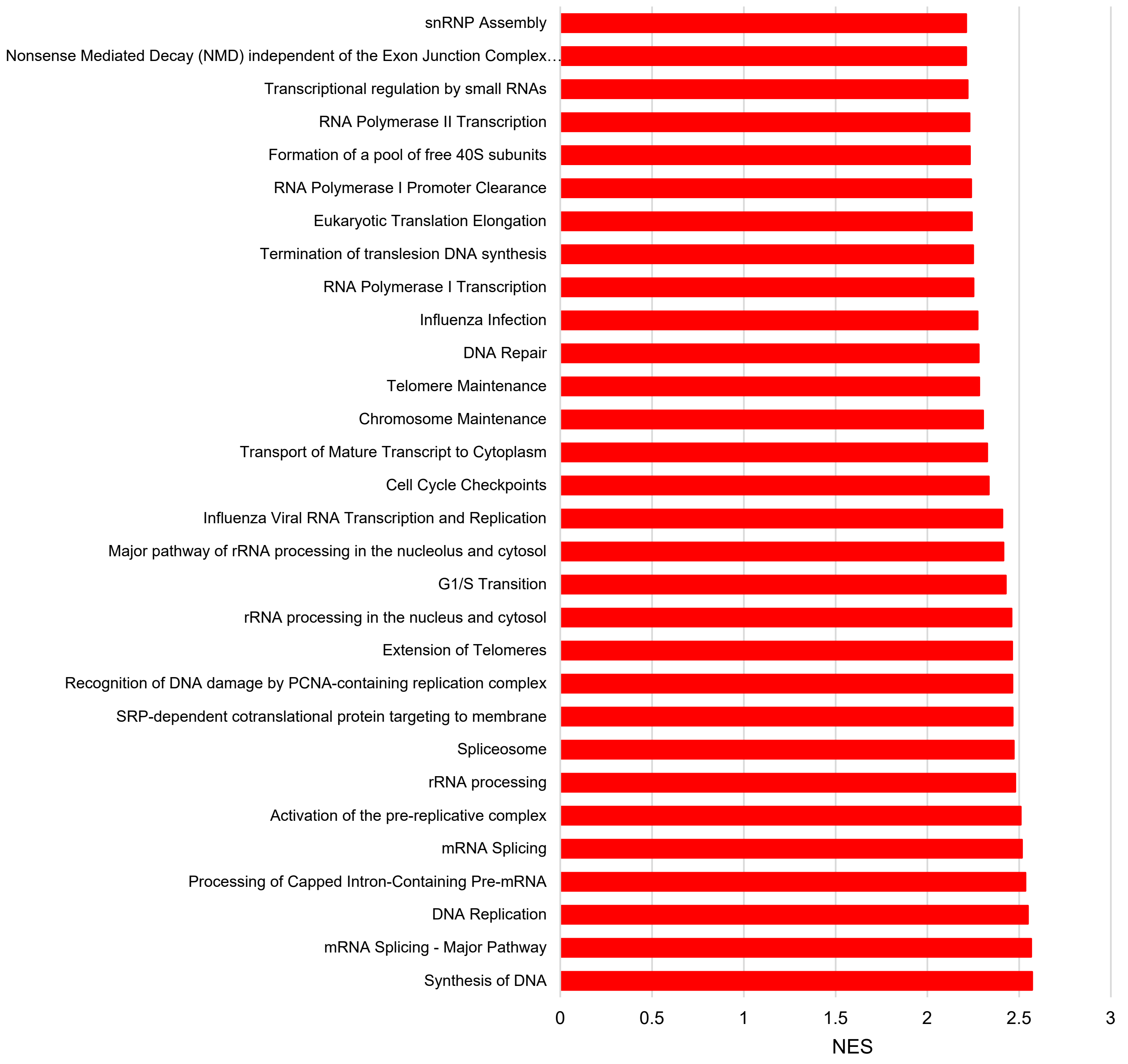

snRNP Assembly
Nonsense Mediated Decay (NMD) independent of the Exon Junction Complex…
Transcriptional regulation by small RNAs
RNA Polymerase II Transcription
Formation of a pool of free 40S subunits
RNA Polymerase I Promoter Clearance
Eukaryotic Translation Elongation
Termination of translesion DNA synthesis
RNA Polymerase I Transcription
Influenza Infection
DNA Repair
Telomere Maintenance
Chromosome Maintenance
Transport of Mature Transcript to Cytoplasm
Cell Cycle Checkpoints
Influenza Viral RNA Transcription and Replication
Major pathway of rRNA processing in the nucleolus and cytosol
G1/S Transition
rRNA processing in the nucleus and cytosol
Extension of Telomeres
Recognition of DNA damage by PCNA-containing replication complex
SRP-dependent cotranslational protein targeting to membrane
Spliceosome
rRNA processing
Activation of the pre-replicative complex
mRNA Splicing
Processing of Capped Intron-Containing Pre-mRNA
DNA Replication
mRNA Splicing - Major Pathway
Synthesis of DNA
0
0.5
1
1.5
2
2.5
3
NES

Supplementary Figure S1. RNAseq-based comprehensive gene expression analysis of HIF-1α knockout human primary NK cells in normoxic conditions compared to hypoxic conditions. (A) Volcano plot analysis. X- and Y-axes: log2 fold change (FC) and log10 P-value, respectively. Red dots: $P < 0.05$. Marked gene expression changes are labeled. (B) Gen set enrichment analysis (GSEA). NES = Normalized enrichment score. GSEA normalizes the enrichment score to account for differences in gene set size and correlations between gene sets and the expression dataset. Selected P-values are $P < 0.01$. Red bars: Upregulated and downregulated gene pathway sets, respectively. The data were computed using RaNAseq (https://ranaseq.eu/index.php).
